# Control of Middle Ear Inflammatory and Ion Homeostasis Genes by Transtympanic Glucocorticoid and Mineralocorticoid Treatments

**DOI:** 10.1371/journal.pone.0119228

**Published:** 2015-03-26

**Authors:** Jessyka G. Lighthall, J. Beth Kempton, Frances Hausman, Carol J. MacArthur, Dennis R. Trune

**Affiliations:** Oregon Hearing Research Center, Department of Otolaryngology, Head & Neck Surgery, Oregon Health & Science University, Portland, OR, 97239-3098, United States of America; University of Ulm, GERMANY

## Abstract

**Hypothesis:**

Transtympanic steroid treatment will induce changes in ion homeostasis and inflammatory gene expression to decrease middle ear inflammation due to bacterial inoculation.

**Background:**

Otitis media is common, but treatment options are limited to systemic antibiotic therapy or surgical intervention. Systemic glucocorticoid treatment of mice decreases inflammation and improves fluid clearance. However, transtympanic delivery of glucocorticoids or mineralocorticoid has not been explored to determine if direct steroid application is beneficial.

**Methods:**

Balb/c mice received transtympanic inoculation of heat-killed *Haemophilus influenzae* (*H flu*), followed by transtympanic treatment with either prednisolone or aldosterone. Mice given PBS instead of steroid and untreated mice were used as controls. Four hours after steroid treatment, middle ears were harvested for mRNA extraction and 24 hours after inoculation middle ears were harvested and examined for measures of inflammation.

**Results:**

*H flu* inoculation caused the increased expression of nearly all inflammatory cytokine genes and induced changes in expression of several genes related to cellular junctions and transport channels. Both steroids generally reversed the expression of inflammatory genes and caused ion and water regulatory genes to return to normal or near normal levels. Histologic evaluation of middle ears showed improved fluid and inflammatory cell clearance.

**Conclusion:**

Improvement in middle ear inflammation was noted with both the glucocorticoid prednisolone and the mineralocorticoid aldosterone. This was due to reversal of inflammation-induced changes in middle ear cytokine genes, as well as those involved in ion and water homeostasis. Because glucocorticoids bind to the mineralocorticoid receptor, but not the reverse, it is concluded that much of the reduction of fluid and other inflammation measures was due to these steroids impact on ion and water transport channels. Further research is necessary to determine if this alternative mineralocorticoid treatment for otitis media will be clinically effective with fewer side effects than glucocorticoids.

## Introduction

Otitis media (OM) is one of the most common infections to affect children.[1] According to the American Academy of Pediatrics Clinical Practice Guidelines, 90% of children will develop at least one episode of otitis media with effusion at some time before school age with a cost estimate of $4 billion annually in the United States.[2] Teele *et al*. reports that 62% of the children experience at least one episode of acute otitis media (AOM) by one year of age and 83% by three years of age, with 17% and 46% having had at least three episodes, respectively.[3] Other authors quote even higher incidences with three or more episodes of acute otitis media afflicting 50% of three year olds and 65% of five year olds.[1] Although many episodes resolve spontaneously, 30–40% have persistent effusion for three months and 5–10% for over one year.[2]

Recurrent acute otitis media (RAOM) and chronic otitis media with effusion (COME) may both cause a conductive or sensorineural hearing loss with developmental sequelae, including impairments in speech or language skills and behavioral changes.[4] Some authors report that the amount of time spent with middle ear effusions before the age of three to be directly correlated to tests of cognitive ability and school performance at age seven years,[5] although this is controversial. Associations between OM and sensorineural hearing loss (SNHL) have been documented.[6] The presence of inflammatory cytokines in the murine inner ear during episodes of AOM and COME has been established and is thought to contribute to hearing loss.[7,8]

Although AOM may clear spontaneously, it is often treated with oral antibiotics to speed recovery, decrease pain, and prevent complications. Conventional medical treatments have shown to be inadequate to effectively treat COME and prevent hearing loss. Oral antimicrobials and corticosteroids have shown to be ineffective in the long-term management of chronic effusions[2] and concerns for the side effects of systemic steroid use have limited their use for these conditions. The American Academy of Otolaryngology-Head and Neck Surgery report individuals with symptomatic RAOM or COME are indicated for surgical drainage via myringotomy with or without placement of a pressure equalizing tube. This requires either an office or operating room procedure and subjects the patient to the risks of tube placement.

Due to the incidence and potential adverse effects of RAOM or COME, research has focused on understanding the pathophysiology of OM, including the role of inflammatory mediators and ion homeostasis molecules. To this end, models using rodents have been developed and are widely used.[9–11] Ion homeostasis proteins have been documented in the middle ear in normal mice and mice receiving a transtympanic inoculation of bacteria.[12] Rodent models have allowed identification of alterations in inflammatory cytokine and ion homeostasis gene expression in both inner and middle ears during AOM and COME.[7,8,13] Studies evaluating oral glucocorticoid steroid treatments during inflammation show a decrease in inflammatory markers, alterations of ion homeostasis genes, and improvements in middle ear inflammation and fluid clearance.[14] Additional research has shown that the genetic changes seen after a bacterial challenge occurs early, with peaks at around six hours after the bacterial challenge.[13] Although the benefits of systemic glucocorticoid use on decreasing inflammation and improving fluid clearance are documented, their use may be limited based on the potential side effects, especially in children. Therefore, other routes of delivery of steroid treatments in an intact tympanic membrane provide an attractive treatment alternative. This study was designed to evaluate the role of transtympanic steroid administration on the early inflammatory changes in response to a bacterial challenge. Furthermore, because ion and water transport channels are under the influence of the mineralocorticoid aldosterone, this steroid also was injected to determine its impact on middle ear inflammation.

## Materials and Methods

All animal procedures in the study were approved by the OHSU Institutional Animal Care and Use Committee

### Steroid control of inflammatory and ion homeostasis genes

#### Animal Model

Balb/c mice were anesthetized with subcutaneous injection of ketamine (100 mg/ml; 0.067 mg/gm) and xylazine (20 mg/ml; 0.013 mg/gm), then screened with otomicroscopy to confirm the absence of middle ear fluid. Inflammation was induced bilaterally by transtympanic injection of 5 μl of heat-killed *Haemophilus influenza* (*H flu*) into the mesotympanum. A minimum of five mice per treatment group was used. Mice subsequently received bilateral injections transtympanically of PBS, prednisolone (10 mg/kg/treatment), aldosterone (0.03 mg/kg/treatment) at either three or six hours after bacterial inoculation. These time-points were selected based on previous research indicating an early alteration in gene regulation after a bacterial insult to the middle ear.[13] Ten normal mice were selected as controls and received bacterial inoculation alone without treatment. Five of the untreated mice were sacrificed at either seven or 10 hours after inoculation to keep time exposure to bacterial challenge consistent with the treatment conditions. An additional 10 mice were not inoculated and served as healthy non-infected controls.

#### Quantitative RT-PCR

Mice were sacrificed four hours after steroid or PBS treatment, the auditory bullae were dissected free of the skull base using microscopic guidance and inner ears were dissected out and discarded. Left and right middle ear tissues were isolated, combined, and stored in RNA*later* (Ambion, Inc., Austin, TX) at -20°C until RNA was extracted. Tissue RNA was extracted with the Qiagen (Valencia, CA) RNeasy Mini Kit. Tissue was transferred to tubes with 600 μl of extraction buffer and homogenized with a PowerGen 125 (Fisher Scientific, Pittsburgh, PA). RNA was quantified using a NanoDrop (Thermo Scientific, Wilmington, DE) and all samples were made up to a concentration of at least 25 ng/μl.

Quantitative real-time PCR studies were performed using an ABI Step One Plus system (Carlsbad, CA). Total RNA (200 ng) was reverse-transcribed using RT^2^ First Strand Kit (SABiosciences Corp, Frederick, MD) using the manufacturer’s instructions. Samples were prepared for real-time PCR using the RT^2^ Real-time SYBR Green/Rox PCR master mix. Thermal cycle condition was set as 95°C 10 minutes then 40 cycles at 95°C for 15 seconds then 60°C for one minute followed by a melt curve. Data analysis follows the suggestion of the manufacturer (SABiosciences PCR Array Data Analysis Web Portal). The parameter CT (threshold cycle) is defined as the fractional cycle number at which the reporter fluorescence generated by cleavage of the probe passes a fixed threshold above baseline. The statistical significance and fold change are calculated using the ΔΔC_t_ method with the aid of SABiosciences PCR Array Data Analysis Web Portal. The housekeeping gene used for this method was glyceraldehyde-3-phosphate dehydrogenase.

This method utilized custom PCR Arrays (SABiosciences Corp, Frederick, MD) already optimized for reaction conditions, primers, and probe. Recent product developments by SABiosciences have combined the two principles of gene arrays and RT-PCR so quantitative RT-PCR can be done simultaneously on primer arrays of multiple cytokine genes (RT^2^ Profiler PCR Array System) for actual quantification of gene expression. This technique has been adapted in our laboratory and a standard cytokine profile is assessed in all inflammatory studies. Our custom PCR Array plates were made by SABiosciences Corp (Frederick, MD) to measure expression of eight key inflammation related cytokines: IL-1α, IL-1β, IL-6, IL-10, MIP-1, MIP-2, KC, and TNFα (Table 1). Additional custom PCR array plates were made to analyze 24 ion homeostasis genes (Table 1). These include isoforms of Na^+^,K^+^-ATPases, channels that transport K^+^, Na^+^ and Cl^-^, gap junction connexins, water transporting aquaporins, and tight junction claudins. These ion homeostasis genes were selected for their known importance in the function of the inner ear and potential role in control of middle ear fluids during inflammation. Gene expression in the inflamed middle ears was compared to non-inoculated and untreated control mice.

**Table 1:** Key inflammatory cytokines and ion homeostasis genes evaluated by PCR array.

Inflammatory cytokine genes:	
**IL-1α, IL-1β, IL-6, IL-10**	interleukins 1 alpha, 1 beta, 6, 10
**MIP-2 (Cxcl2), MIP-1 α (CCL3), KC (Cxcl1)**	chemokines
**TNFα**	tumor necrosis factor α
**Ion Homeostasis Genes:**	
**Aqp1, 2, 3, 5**	aquaporins 1, 2, 3, 5
**Atp1β 1, Atp1β2**	Na+,K+-ATPase, Na+/K+ transporting, beta 1, 2, polypeptides
**Atp1α1**	Na+,K+-ATPase, Na+/K+ transporting, alpha 1 polypeptide
**Clcnka**	chloride channel Ka
**Cldn3, 4, 14**	claudins 3, 4, 14
**Gja1, Gjb2, 3, 6**	gap junction proteins, alpha 1, beta 2, 3, 6
**Kcne1, Kcnq 1, 4**	potassium voltage-gated channels
**Kcnj10**	potassium inwardly-rectifying channel
**Slc12a2 (Formerly NKCC1)**	Na+-K+-2Cl- co-transporter
**Scnn1α, 1β, 1γ**	ENaC (epithelial Na+ channels), non-voltage gated 1α, 1β, 1γ
**Tmprss3**	transmembrane protease, serine 3

### Steroid control of inflammatory changes and fluid accumulation in the middle ear

Mice were anesthetized with subcutaneous injection of ketamine and xylazine then screened with otomicroscopy for the absence of middle ear fluid. Inflammation was induced in 20 mice by inoculation with bilateral transtympanic injection of 5μl of heat-killed *H flu*. A minimum of five mice per treatment group subsequently received bilateral transtympanic PBS, prednisolone, or aldosterone at either three or six hours after bacterial inoculation. Five mice received bacterial inoculation alone with no steroid treatment. Based on standard procedure in our laboratory, mice were euthanized 24 hours after inoculation and intracardially perfused with fixative of 1.5% glutaraldehyde and 3% paraformaldehyde in 0.1 mol/L phosphate buffer. Skulls were dissected with the auditory bullae left intact such that both ears were connected to each other by the skull base. Dissected skulls were immersed in fixative overnight then processed for histology and sectioning. Tissues were microwave decalcified in EDTA, embedded in glycol methacrylate plastic, sectioned in the horizontal plane at 5 μm, serially mounted on glass slides, stained with basic fuchsin and methylene blue, and cover-slipped. Slides were examined at 10 x magnification with the Leica DMLB microscope. Middle ears were qualitatively evaluated for fluid area, inflammatory cell infiltration, tympanic membrane thickness, and mucosal thickness based on previous research identifying these measures to best assess inflammation.[10] Grading of the above parameters was performed with blinding to treatment condition using a calibrated micrometer grid in one eyepiece and a micrometer scale in the opposite eyepiece. A minimum of five mice per condition were evaluated and each ear was examined separately. Three sections per middle ear were selected and standardized to include the tympanic membrane and round window at the level of the stapedial artery. The three individual measures per ear were averaged to obtain one value for each parameter per ear. Statistical analyses using analysis of variance and Tukey post-hoc tests (SPSS Inc, Chicago, Illinois) were performed comparing treatment groups to control mice who received neither inoculation nor treatment and mice who received inoculation alone with no treatment.

## Results

### Bacterial effect on middle ear gene expression

**Fig 1:**
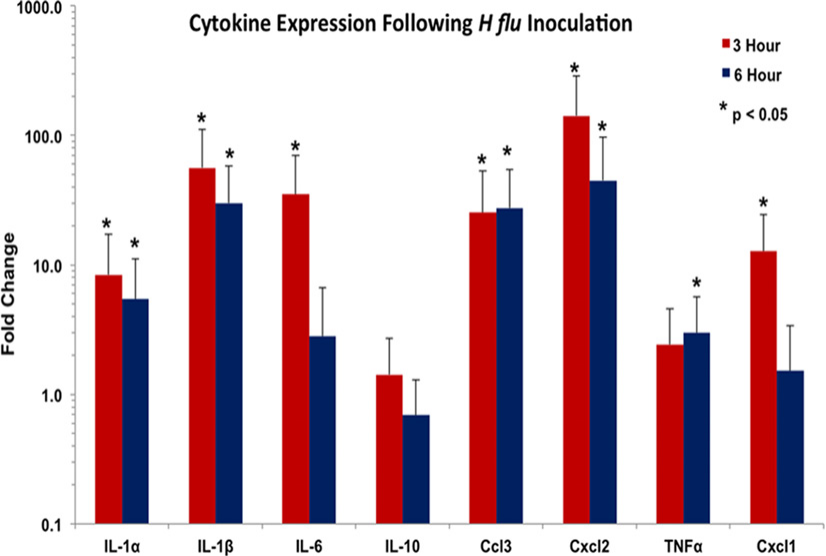
Cytokine gene expression for the control animals that received no treatment three hours and six hours after transtympanic inoculation with *H flu*. This reflects fold change in gene expression seven and ten hours, respectively, after inoculation compared to animals receiving no bacteria. All inflammatory cytokine genes were significantly upregulated with the exception of IL-10 and TNFα. By ten hours, most were still elevated, although the trend was for their gradual decline in the overexpression seen at the earlier time point.

The inoculation of heat-killed bacteria in to the middle ear caused significant changes in the expression of multiple genes, some related to cytokines and some involved in ion and water transport. Every pro-inflammatory gene was upregulated after three hours, with most still showing increased expression at six hours (Fig. 1). This elevated gene activity was especially pronounced for the interleukins and chemokines. IL-1β was expressed at 56 times normal levels, IL-6 was overexpressed by 35-fold, and Ccl3 (MIP-1α) and Cxcl2 (MIP-2) were expressed at 25 and 141 times normal levels, respectively. These same inflammatory genes were still significantly elevated at six hours, although their levels had begun to decline (Fig. 1). The anti-inflammatory IL-10 was not significantly affected at either time point.

**Fig 2:**
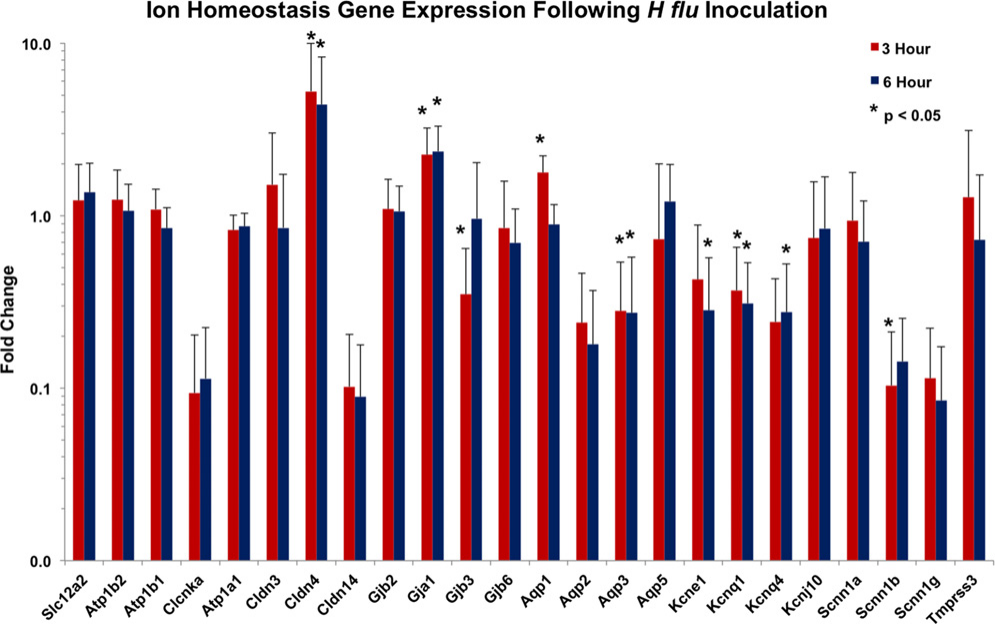
Ion homeostasis gene expression for the control animals that received no treatment three hours and six hours after transtympanic inoculation with *H flu*. This reflects fold change in gene expression seven and ten hours after inoculation compared to animals receiving no bacteria. Although there were some genes that were expressed at higher than normal levels (claudin 4, gap junction a1, aquaporin 1), the majority of homeostasis genes in the middle ear were suppressed in expression. Many other genes were reduced to 10–30% of normal expression (Clcnka, claudin 14, aquaporins 2 and 3, most postassiumchannels, and ENac channels Scnn1b and Scnn1g), but these did not reach statistical significance due to variability. In general, the expression levels seen in the three hour controls were similar to those in the six hour controls.

The inoculation of the middle ear also caused considerable alteration in the expression of several ion homeostasis genes (Fig. 2). Some genes were overexpressed, while others showed decreased expression. The tight junction claudin 4 gene measured over five-fold normal expression, gap junction gene Gja1 was over two times normal activity, and the Aqp1 water channel was up-regulated 1.8-fold (Fig. 2). However, generally these ion homeostasis genes were down-regulated due to the local inflammation. Significant reductions were seen for the water channel Aqp3, the K^+^ transporting channels Gjb3, Kcnq1, and Kcnq4, and the epithelial Na^+^ channel Scnn1g. Also, several other junction, channel, or transporter genes were expressed at reduced levels (Clcnka, Cld14, Aqp2, Kcne1, Scnn1b), but these did not reach the statistical cutoff of p < 0.05. As with the cytokines, recovery to normal levels of expression was seen for several of these channels by six hours (Fig. 2). These measured changes reflect the degree to which the middle ear mucosa reacts to bacteria and is the basic tissue profile on which to evaluate the impact of steroids.

### Steroid effect on middle ear gene expression

**Fig 3:**
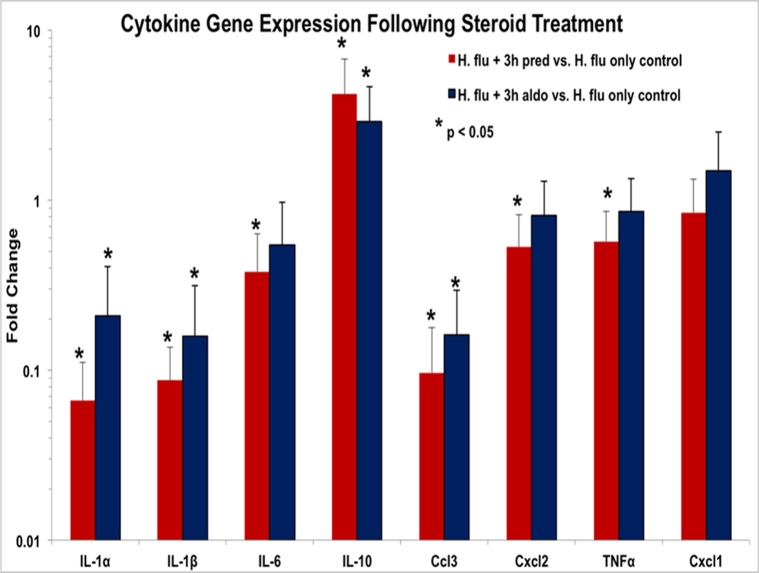
Impact of steroids on cytokine gene expression. Mice received either prednisolone (Pred) or aldosterone (Aldo) three hours after after transtympanic inoculation with *H flu* and harvested four hours later (total seven hours post inoculation). Expression of several of the cytokine genes was reduced compared to *H flu* only mice shown in Fig. 1. In general, both Pred and Aldo were similar in their suppression of the genes impacted by the bacteria. Although a few genes showed significant effects with Pred and not Aldo (IL-6, Cxcl2, TNFα), the differences in effects of the two steroids were minimal.

Transtympanic treatment with mineralocorticoid or glucocorticoid induced significant alteration of middle ear inflammatory gene expression. When given three hours after bacterial inoculation, prednisolone induced down-regulation of pro-inflammatory and up-regulation of anti-inflammatory (i.e. IL-10) cytokine genes (Fig. 3). Aldosterone treatment also resulted in a down-regulation in several inflammatory cytokine genes and an increase in IL-10 (Fig. 3). The glucocorticoid significantly reduced six of the overexpressed genes, while aldosterone reduced only three of them. This suggests the glucocorticoid, with its strong anti-inflammatory effects, was more effective, although the difference between the two steroids was minor. When steroids were administered six hours after inoculation, prednisolone showed a persistent increase in IL-10, as well as up-regulation of the pro-inflammatory cytokines IL-1β, IL-6, Cxcl-2, and Cxcl-1. Only TNFα was suppressed. On the other hand, aldosterone treatment at six hours did not show any increased expression of cytokines compared to no treatment, and also caused a decrease in TNFα (results not shown). Thus, waiting to administer steroids until six hours had little immune suppressive effect.

**Fig 4:**
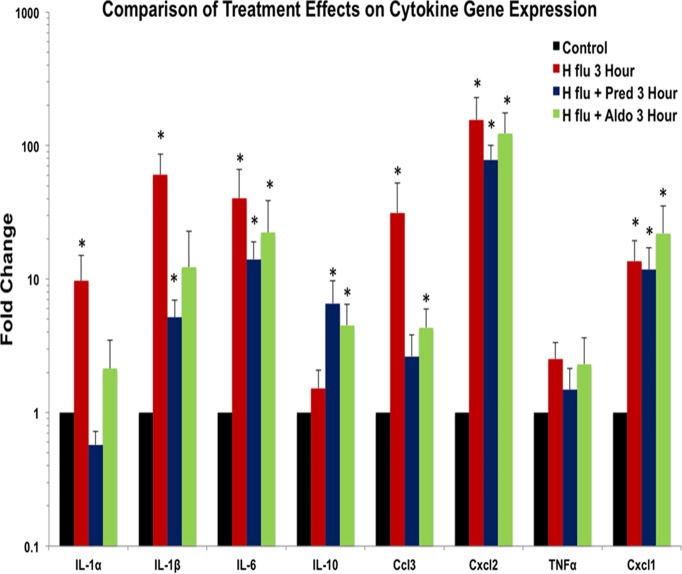
Comparison of treatments on cytokine gene expression. A comparison was made of untreated controls, *H flu* only mice, and the two steroid treatments to determine how effective the steroids were is returning inflammatory gene expression back to a normal level. When expression fold change is compared to untreated normal mice, a general pattern is seen of steroids reducing overexpression of inflammatory genes due to *H flu* back to levels near that of untreated normal mice. Only IL-6, IL-10, Cxcl2, and Cxcl1 maintained their overexpression levels due to H flu and were not reduced by steroids. The remaining cytokines showed no difference from normal following one or both of the steroids.

While steroid treatments reduced the expression of inflammatory cytokine genes, it was not clear if such treatments reduced their activity back to normal levels. Therefore, a statistical comparison was made of normal mice relative to the H flu only mice and the two steroid treatments for their respective 3 hour results (Fig. 4). The single transtympanic steroid treatment brought expression levels back to near normal for several of the cytokines. IL-1α expression returned to normal with both steroids as neither was statistically different from the control mice. IL-1β was but still slightly elevated after both steroids, but only the glucocorticoid treatment was still statistically significant. IL-6 did not respond well to steroids as neither steroid reduced the elevated expression levels from bacteria. Also IL-10, an anti-inflammatory cytokine, was significantly elevated beyond normal expression by both steroids. Ccl3 (Mip-1α) expression was returned to near normal levels by the steroids. The remaining cytokines (Cxcl2, TNFα, Cxcl1) did not show a return to normal levels by the steroid treatments. Thus, while all cytokines generally showed significant up-regulation due to *H flu*, some did not return to normal levels of expression with the steroid treatments.

**Fig 5:**
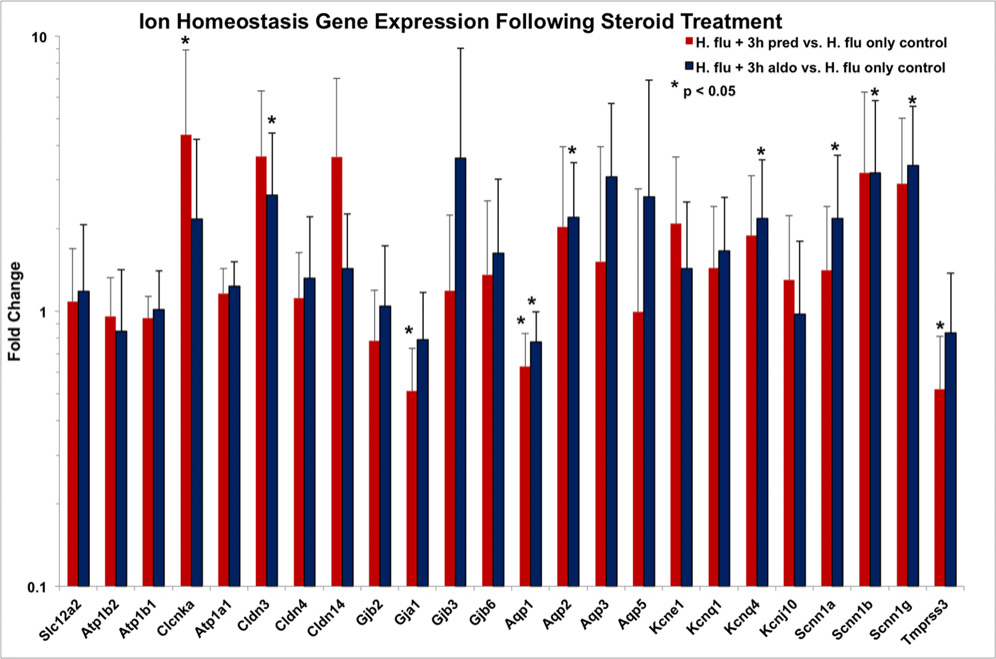
Impact of steroid treatment on ion homeostasis gene expression. Mice received either prednisolone (Pred) or aldosterone (Aldo) three hours after after transtympanic inoculation with *H flu* and harvested four hours later (total seven hours post inoculation). With the exception of Gja1, Aqp1, and Tmprss3, most of the genes controlling ion and fluid balances were upregulated by the steroids.

Administration of mineralocorticoid or glucocorticoid had significant effects on middle ear ion homeostasis gene expression, as well. When given three hours after inoculation, prednisolone induced a down-regulation of gene expression in a gap junction, aquaporin, and epithelial sodium channel-related molecule (Tmprss3) when compared to untreated controls (Fig. 5). Conversely, aldosterone induced gene expression of multiple ion homeostasis molecules, including subunits of claudins, aquaporins, potassium channels and epithelium sodium channels (Fig. 5). In general, ion homeostasis genes were up-regulated by aldosterone when it was given after bacterial inoculation. When administered six hours after bacterial inoculation, little effect on ion homeostasis gene regulation was noted (data not shown).

**Fig 6:**
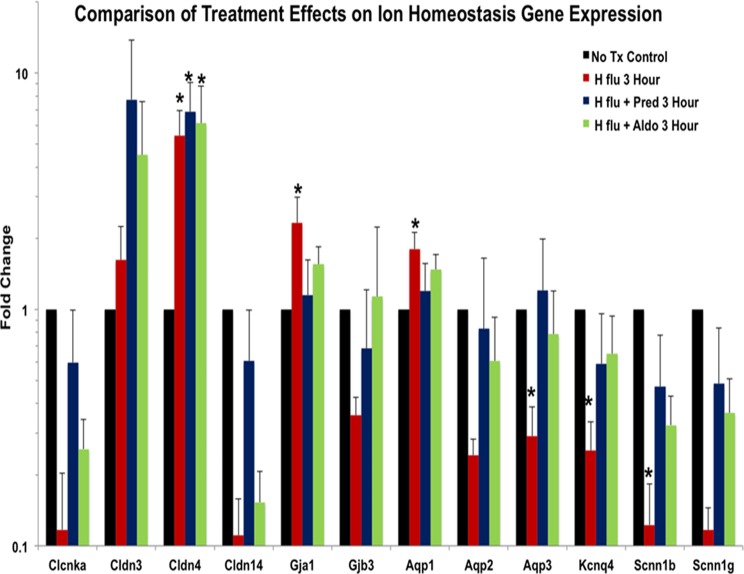
Comparison of treatments on cytokine gene expression. A comparison was made of untreated controls, *H flu* only mice, and the two steroid treatments to determine how effective the steroids were is returning homeostasis genes back to a normal level. Claudin 4 was upregulated by *H flu* and its levels were not reduced by the steroids. The remaining genes were significantly up or down regulated by the *H flu* treatment, and steroid treatments returned expression of these genes back to normal levels.

When untreated, H flu only, and H flu plus steroid mice were compared, a quite consistent pattern was seen for the ion homeostasis genes (Fig. 6). Generally, any change in gene expression induced by the bacteria was reversed by the steroids. This is particularly noted for Clcnka, Cldn14, Gjb3, Aqp2 and 3, Kcnq4, and the epithelial Na^+^ channels Scnn1b and Scnn1g. All of these genes were dramatically suppressed by the middle ear inflammation. This suppression was reversed by the steroid treatments so their expression was generally not different from the untreated control mice. There was little difference observed between the aldosterone and prednisolone, suggesting both had comparable impact on these genes. A few middle ear homeostatic genes were up-regulated by the inflammation, and this up-regulation was reversed by the steroids. This was seen for Gja1 and Aqp1 (Fig. 6). However, the general pattern seen was suppression of water and ion transport genes by the inflammation and restoration of expression to normal levels by the steroid treatments.

### Steroid effect on middle ear histopathology

**Fig 7:**
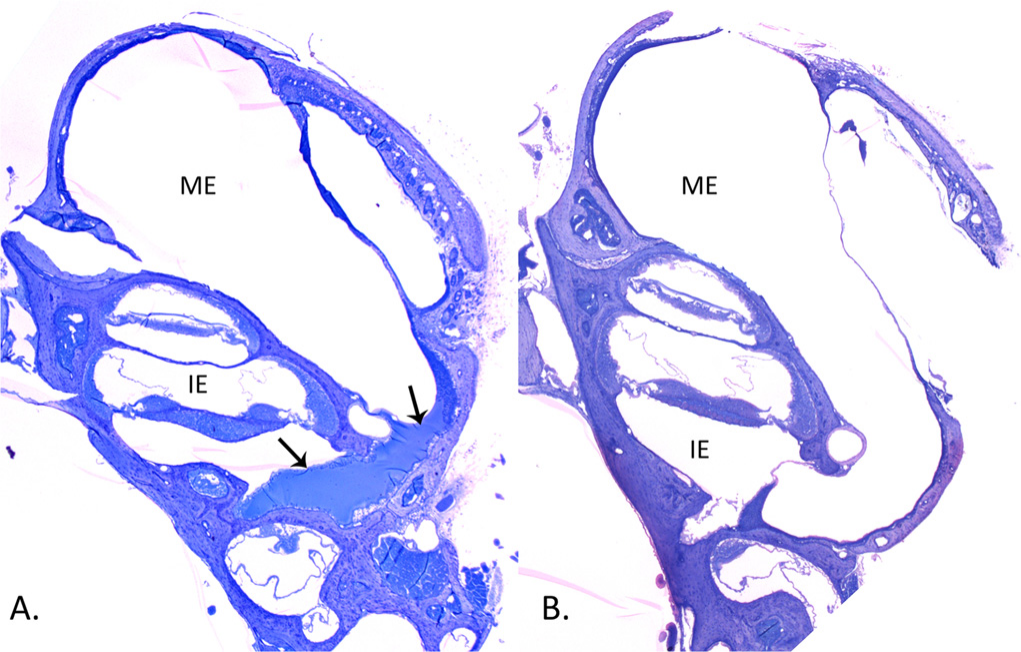
Histologic comparison of the murine middle ear 24 hours after inoculation with *H flu* before (A) and after (B) glucocorticoid treatment. **A:** Note the extensive fluid (arrows) in the middle ear (ME) space, particularly against the round window membrane (left arrow) adjacent to the inner ear (IE) scala tympani **B:** The murine middle ear 24 hours after inoculation with *H flu* and treatment with transtympanic prednisolone three hours later. The middle ear is nearly clear of fluid except for a small amount against the round window membrane.

Quantitative analysis of middle ears showed that PBS was not different from H flu only treatments in all measures. Thus, differences seen with the steroid treatments are presumed to be due to their specific impact rather than vehicle. Prednisolone, when given three or six hour after inoculation, caused a significant decrease in tympanic membrane thickness (Table 2). It also significantly decreased fluid in the middle ear when it was administered six hours after bacterial inoculation, although the probability for the three hour treatment was p = 0.0535, very close to the cutoff for significance. Histologic examination for the three hour treatment showed dramatic reduction in fluid within the middle ear space (Fig. 7). The number of inflammatory cells was significantly decreased by prednisolone at both three and six hours (Table 2). Mucosal thickness was not improved by prednisolone at three hours; however it was following treatment at six hours (Table 2).

**Table 2:** Comparison of histologic markers of middle ear inflammation when transtympanic prednisolone (Pred) or aldosterone (Aldo) were given three (+ 3h) or six (+ 6h) hours after inoculation with *H flu* compared to no-treatment controls. ANOVA compared all five treatment groups for each measure and the respective F ratio and probability are listed for each quantitative measure. All for quantitative measures showed a difference among the treatments groups so Post-Hoc tests were run to determine the statistical differences between the individual steroid treatment groups and H flu only controls. Those statistically significant are in bold.

				Tukey Post-Hoc
Control	Treatment	Mean	Std Error	Significance
**Fluid Area (μm^2^): F = 4.96, p = 0.003**				
	*H*.*flu* only	5602.1	710.6	
	*H flu* + 3h Pred	2709.3	961.5	0.0535
	*H flu* + 3h Aldo	2321.1	961.5	**0.0176**
	*H flu* + 6h Pred	2576.0	832.7	**0.0090**
	*H flu* + 6h Aldo	1196.8	961.5	**0.0004**
**Total # Inflammatory Cells: F = 9.286, p = 0.00000017**				
	*H*.*flu* only	1227.7	225.4	
	*H flu* + 3h Pred	396.0	176.6	**0.000235**
	*H flu* + 3h Aldo	348.7	176.6	**0.000087**
	*H flu* + 6h Pred	407.7	153.0	**0.00002**
	*H flu* + 6h Aldo	235.7	176.6	**0.000007**
**TM Thickness (μm): F = 9.408, p = 0.0000001**				
	*H*.*flu* only	0.2573	0.02	
	*H flu* + 3h Pred	0.1453	0.0217	**0.00004**
	*H flu* + 3h Aldo	0.1893	0.0217	**0.03862**
	*H flu* + 6h Pred	0.1480	0.0188	**0.000003**
	*H flu* + 6h Aldo	0.1320	0.0217	**0.000004**
**Mucosal Thickness (μm): F = 4.505, p = 0.001**				
	*H*.*flu* only	1.507	0.606	
	*H flu* + 3h Pred	1.120	0.1528	0.164
	*H flu* + 3h Aldo	1.080	0.1528	0.092
	*H flu* + 6h Pred	0.860	0.1324	**0.0001**
	*H flu* + 6h Aldo	0.960	0.1528	**0.011**

Aldosterone treatment resulted in a significant reduction in fluid area, number of inflammatory cells, as well as thickness of the tympanic membrane when transtympanically administered either three or six hours after bacterial inoculation (Table 2). Fluid area was dramatically decreased at six hours, reaching levels less than half that seen with prednisolone treatment. A similar improvement over prednisolone at six hours was noted for the number of inflammatory cells, which were seen in reduced number after aldosterone treatment (Table 2). The effect of aldosterone on TM and mucosal thickness also was significant. Like prednisolone, mucosal thickness was improved after the six hour treatment only (Table 2). Thus aldosterone was either equally effective or more effective compared to prednisolone in these quantitative measures. This indicates that the increased expression of numerous ion homeostasis genes above resulted in clearance of inflammatory components and measures. Post-hoc tests showed no statistical difference between prednisolone and aldosterone, indicating they were quantitatively comparable in their inflammation clearing effects.

## Discussion

### Inflammatory Cytokines

This study profiled the early cytokines produced in the middle ear following H flu inoculation. Several inflammatory cytokines were up-regulated 10–100 fold by three hours, including IL-1α, IL-1β, IL-6, Ccl3 (MIP-1α), Cxcl2 (MIP-2), and Cxcl1 (KC). Furthermore, steroid treatment with either prednisolone (glucocorticoid) or aldosterone (mineralocorticoid) resulted in decline of this cytokine overexpression. It did appear that the glucocorticoid, with its direct immune suppression function, was slightly more effective in reducing the cytokine expression back to normal, but the mineralocorticoid aldosterone was nearly as effective. By waiting to apply the steroids until six hours, their effect was lessened. This suggests the inflammatory process is more advanced by that time and immune suppressive glucocorticoids cannot overcome the advanced stage of inflammatory processes. Interestingly, the aldosterone treatment at six hours had a more suppressive effect than the traditional glucocorticoid, as it did not allow further increased expression of the cytokine genes. The fact aldosterone does not bind to the glucocorticoid receptor suggests it kept inflammatory cytokines from increasing due to its impact on fluid control. This will be addressed below under ion and homeostasis impact.

These early results of middle ear inflammation parallel findings in other laboratories. In an extensive review of inflammatory mediators in otitis media by Juhn *et al*.[15] early phase cytokines include IL-1 and TNFα, both of which are produced by both inflammatory cells and local epithelial cells in the middle ear. IL-1 induces further inflammation by stimulating other cytokines, additional IL-1 production (positive feedback loop), and arachidonic acid metabolism. Because of these functions, early IL-1β has been shown to play a key role in the development of acute OM and middle ear effusions,[16–19] similar to findings here. More recently, MacArthur *et al*.[13] found that after middle ear injection of H flu, the peak expression of MIP-2, IL-1β, IL-6, KC, and IL-10 occurred at six hours after inoculation. TNFα and MIP-1α also increased at six hours, but peaked at 24 hours, and IL-1α peaked at six hours and remained elevated at 24 hours. Levels of all of these markers were decreasing by 24 to 72 hours post-inoculation. The present findings of early phase cytokine profiles, and their suppression by steroids, suggest that therapies targeted at the early stages of infection may abrogate the development of OM.

### Ion & water transport

Currently little is known of the function of these various ion and water channels in the middle ear. All occur in the inner ear where they are mainly involved in the blood labyrinth barrier and moving ions or water to establish the endolymph for the critical endocochlear potential (high potassium, low sodium).[20–22] However, the natural state of the middle ear is fluid free, so their homeostatic roles must be different. Morris *et al*.[12] localized many of these same ion channels and transporters, aquaporin water channels, and intercellular junctional proteins in the normal and inflamed murine middle ear epithelium using immunohistochemistry. Other studies have demonstrated that these ion channels, such as the epithelial sodium channel, are responsible for keeping the middle ear clear of fluid.[23]

Although the accumulation of middle ear fluid is multi-factorial, transepithelial ion and water transport mechanisms are affected by bacterial inoculation[12,13,24] and may play a significant role in the development and persistence of middle ear effusions. Several studies have noted the interplay between inflammation and ion or fluid homeostasis. Specifically, IL-1β or whole bacteria have been shown to play a key role in the development of acute OM and middle ear effusions by up-regulating the Na-K-2Cl cotransporter and suppressing the sodium channel genes.[24–27] Thus, a decrease in sodium transport could lead to a decrease in fluid clearance. Because both glucocorticoids and mineralocorticoids impact many of these ion homeostasis genes by binding to the mineralocorticoid receptor,[28] their application for otitis media in the present study presumably improved clearance of middle ear fluid and cells. It is particularly noteworthy here that the mineralocorticoids, which have not been used before experimentally for OM, were as effective as glucocorticoids. Aldosterone induced expression of multiple ion homeostasis genes, including subunits of claudins, aquaporins, potassium channels and epithelium sodium channels. Similar studies of glucocorticoids for OM have suggested they operate by improving sodium ion transport functions.[29,30] Thus, the presumed function of both steroids in clearing middle ear fluids is the mineralocorticoid receptor mediated up-regulation of sodium channel functions.

MacArthur *et al*.[31] evaluated the role of systemic steroid treatment in middle ear inflammation and sensorineural hearing loss in C3H/HeJ mice with spontaneous chronic otitis media. Mice showed improvement in ABR with both systemic mineralocorticoid (aldosterone) and glucocorticoid (prednisolone, dexamethasone) treatment and histologic improvement in middle and inner ear inflammation in the group treated with prednisolone. In a parallel acute OM study, MacArthur *et al*.[14] examined the middle ear of Balb/c mice after transtympanic inoculation with S*treptococcus pneumoniae*, followed by systemic treatment with glucocorticoid or mineralocorticoid starting on the day prior to inoculation. Mice were taken at three and five days after inoculation and middle and inner ears were examined histologically for inflammation. Although glucocorticoids were the most effective at decreasing tympanic membrane thickness at three days, both glucocorticoids and mineralocorticoids were effective in suppressing middle ear inflammation at five days. These various findings suggest therapies targeting expression these ion homeostasis genes may be integral in the treatment of effusions.

This study has clear limitations. First, it utilizes a murine model, and although these have been shown to be useful for studying otitis media, the effects seen may not necessarily be the same in human subjects. In addition, this study uses heat-killed bacteria that also may not mimic human otitis media as mammalian models of otitis media utilizing live bacteria have shown inconsistent and variable results. Third, this study is aimed at evaluating the basic molecular mechanism of action of steroids on inflammatory and ion homeostasis genes in the middle ear after a bacterial challenge. It is not intended to mimic a true clinical situation at this time and additional research using later post-inoculation treatment times and studies with other murine models that develop spontaneous otitis media would be useful. Despite these limitations, these findings suggest that transtympanic steroid administration for either acute or chronic otitis media may present a viable treatment option and should be studied further. Critical future studies would ideally identify the specific ion and water transport processes that clear middle ear inflammation following treatment with transtympanic mineralocorticoids and glucocorticoids.

## Conclusions

Transtympanic steroids provide an attractive alternative therapy for acute and chronic otitis media. This study shows that when administered early after a bacterial challenge to the middle ear, both glucorticoids and mineralocorticoids cause a decrease in pro-inflammatory gene expression, increase in anti-inflammatory gene expression, and improvement in histologic findings of fluid accumulation and inflammation occur. However, additional research is necessary to further elucidate the mechanism of action and the preferential utility of either treatment for acute and chronic otitis media prior to human studies.
